# Molecular cloning and functional characterization of BcTSA in the biosynthesis of indole alkaloids in *Baphicacanthus cusia*

**DOI:** 10.3389/fpls.2023.1174582

**Published:** 2023-04-17

**Authors:** Zhiying Guo, Junfeng Chen, Zongyou Lv, Yuxiang Huang, Hexin Tan, Lei Zhang, Yong Diao

**Affiliations:** ^1^School of Food and Bioengineering, Fujian Polytechnic Normal University, Fuqing, China; ^2^School of Medicine, Huaqiao University, Quanzhou, China; ^3^Institute of Chinese Materia Medica, Shanghai University of Traditional Chinese Medicine, Shanghai, China; ^4^School of Pharmacy, Navy Medical University, Shanghai, China

**Keywords:** *Baphicacanthus cusia*, indole alkaloids, tryptophan synthase alpha-subunit, molecular cloning, functional characterization

## Abstract

*Baphicacanthus cusia* (Nees) Bremek (*B. cusia*) is an essential traditional Chinese herb that is commonly used to treat colds, fever, and influenza. Indole alkaloids, such as indigo and indirubin, are the primary active constituents of *B. cusia*. The indole-producing reaction is crucial for regulating the flow of indole alkaloids metabolites along the pathways and coordinating primary and secondary product biosynthesis in plants. The tryptophan synthase alpha-subunit (TSA) can catalyse a process that produces indole, which is free to enter secondary metabolite pathways; however, the underlying potential mechanism of regulating indigo alkaloids synthesis remains unknown. Here, a *BcTSA* was cloned from the transcriptome of *B. cusia*. The BcTSA has a significant degree of similarity with other plant TSAs according to bioinformatics and phylogenetic analyses. Quantitative real-time PCR (RT-qPCR) research showed that *BcTSA* was dramatically enhanced in response to treatment with methyl jasmonate (MeJA), salicylic acid (SA), and abscisic acid (ABA), and was predominantly expressed in the stems as opposed to the leaves and rhizomes. Subcellular localization revealed that BcTSA is localized in chloroplasts, which is compatible with the fact that the conversion of indole-3-glycerol phosphate (IGP) to indole occurs in chloroplasts. The complementation assay results showed that BcTSA was functional, demonstrating that it was capable of catalyzing the conversion of IGP to indole. *BcTSA* was shown to stimulate the manufacture of indigo alkaloids including isatin, indigo, and indirubin when the gene was overexpressed in the hairy roots of *Isatis indigotica*. In conclusion, our research provides novel perspectives that might be applied to manipulating the indole alkaloid composition of *B. cusia*.

## Introduction

*Baphicacanthus cusia* is an important medicinal plant in the family of Acanthaceae that is extensively distributed in Southwest China, South China, and East China. Its roots are used as a valuable medication known as “Nan-Ban-Lan-Gen”, which is classified in the Chinese Pharmacopoeia. *Indigo naturalis* (Qingdai), is a blue powder extracted from the leaves and stems of *B. cusia* plants using fermentation (Huang et al., 2017; Zeng et al., 2018). To date, several chemicals from *B. cusia* have been isolated and identified, including indole alkaloids, quinazolinone alkaloids, flavonoids, monoterpenes, triterpenes, sterols, anthraquinones, and benzoxazinones (Li et al., 1993; Gu et al., 2014; Feng et al., 2016; Lee et al., 2019). The main active pharmaceutical ingredients of *B. cusia* are known to be indole alkaloids such as indigo and indirubin (Huang et al., 2014; Hu et al., 2021).

Indigo (also known as indigotin) exhibited anti-inflammatory activities *in vitro* (Li et al., 2022). Indigo, is also a dye that has been cherished since antiquity for its brightness and deep blue color and has also been used for 6,000 years. Indigo, unlike most dyes, adsorbs rather than covalently binds to cotton fibers. The adsorbed indigo is resistant to harsh laundry detergents. Still, it peels off with repeated abrasion to reveal the interior white yarn core, resulting in the desirable worn-in effect that personalizes a pair of jeans. Indigo’s unique mix of resistance to detergents and abrasion makes it irreplaceable as a denim dye and adds to the lasting appeal of blue denim (Warzecha et al., 2007; Hsu et al., 2018; Xu et al., 2020). Despite the fact that synthetic indigo is more affordable, indigo has a great deal of commercial potential since it has accessory pigments (indigo, isoindigo, indirubin, isoindigotin, indigo gluten, and indigo yellow), which make natural indigo more attractive than synthetic indigo (Maugard et al., 2001). Indirubin (isoindigotin or indigo red), a bis-indole alkaloid, is the active constituent of the Chinese patent medicines “Huang Dai Tablets” and “Danggui longhui wan”, which is considered to have an anti-tumor effect and is clinically used in the treatment of chronic myelocytic leukaemia (CML), inhibits cyclin-dependent kinases (Hoessel et al., 1999; Wang et al., 2008; Wang et al., 2021). However, these chemicals are found in low natural quantities in *B. cusia*. As a result, increasing the concentration of active metabolites is important for improving *B. cusia* quality while also meeting market demands.

**Figure 1:**
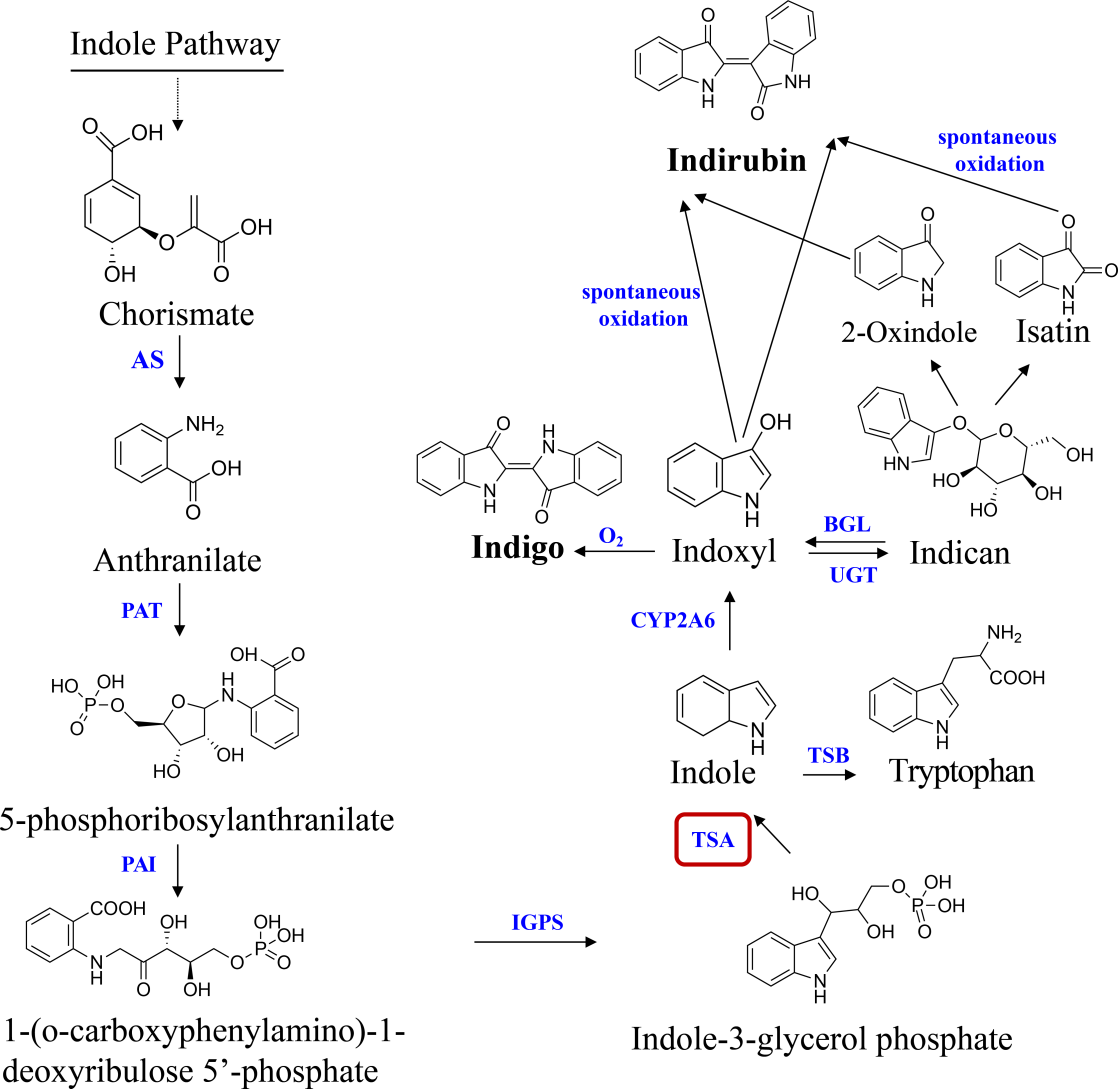
Pathway of indole alkaloids biosynthesis in *B* *cusia*. AS, anthranilate synthase; PAT, phosphoribosyl anthranilate transferase; PAI, phosphoribosyl anthranilate isomerase; IGPS, indole-3-glycerol phosphate synthase; TSA, tryptophan synthase alpha-subunit; TSB, tryptophan synthase beta-subunit; CYP2A6, cytochrome P450 monooxygenase; BGL, β-glucosidase; UGT, UDP-glucosyltransferase. Solid lines indicate a single step; dotted lines indicate multiple steps.

In the past 30 years, indole alkaloids biosynthesis has been among the most intensively investigated secondary metabolic pathways in blue-genera plants, such as *Isatis indigotica* (Salvini et al., 2008), *Polygonum tinctorium* (Inoue et al., 2018) and *B. cusia* plants (Hu et al., 2021), etc. Presently, the potential pathway of indole alkaloids biosynthesis is given as follows: chorismate is catalyzed to create anthranilate by anthranilate synthase (AS). Phosphoribosyl anthranilate transferase (PAT), phosphoribosyl anthranilate isomerase (PAI), and indole-3-glycerol phosphate synthase (IGPS) function sequentially to further convert anthranilate to indole-3-glycerol phosphate (IGP). The conversion of IGP and serine to tryptophan is catalysed by the tryptophan synthase (TS, EC 4.2.1.2) complex. TS is a heterotetramer composed of two alpha-(TSA) and two beta-(TSB) subunits (Salvini et al., 2008). In some plant species, TSA-related proteins also exist that, without binding TSB, transform IGP into glyceraldehyde 3-phosphate (G3P) and indole. Indole was oxygenated by cytochrome P450 monooxygenase (CYP2A6) to create highly reactive indoxyl, which was subsequently glucosylated *in vivo* by UDP-glucosyltransferase (UGT) (Xu et al., 2020). When a plant is harmed, the vacuole compartment vanishes, and the indican in the vacuole is converted to indoxyl by beta-glucosidase (GLU) in the chloroplast. Indoxyl is subsequently oxidized by the oxygen in the air to dimerize and generate blue indigo. Indirubin is created by the condensation of isatin and indoxyl, which occurs when the two substances combine (**Figure 1**). Due to (i) the instability of indoxyl, the catalytic biosynthesis of indigo and indirubin is extremely complex (Maugard et al., 2001); (ii) the plant CYP family, which is normally classified as a monooxygenase, is important for the biosynthesis pathways of secondary metabolisms. However, they catalyze a wide range of reactions and have a low degree of sequence similarity (Xu et al., 2020). As a result, it is still not entirely known what distinct evolutionary processes and molecular mechanism underlie the indigo and indirubin biosynthesis pathways in plants.

In plants, indole is a key precursor for secondary metabolites that have multiple functions. We, therefore, proposed that TSA is one of the essential enzymes that catalyze indole synthesis in the biosynthesis of indole alkaloids. The present study not only systematically identified *BcTSA* by analyzing the *B. cusia* transcriptome sequence using a set of bioinformatics approaches, and characterized its sequence features, exon-intron structures, phylogenetic relationship, and expression profiles, but also verified the biological functions of BcTSA in the regulation of indole alkaloids biosynthesis by a series of *in vivo* and *in vitro* assays. Taken together, our results provide new insights into the role of *BcTSA* in the biosynthesis of indole alkaloids, as well as a solid theoretical experimental foundation for acquiring high-yielding indole alkaloids and optimizing *B. cusia* breeding in the future.

## Materials and methods

### Plant materials and treatments

*B. cusia* plants were grown in perennial dominant Shufeng Farm in Fujian province, China (25°25 N 118°39 C). *I. indigotica* plants were cultivated in the Naval Medical University’s (NMU) experimental field in Shanghai, China. Professor Hanming Zhang (School of Pharmacy) conducted the species verification. Six-month-old *B. cusia* plants were subjected to hormone treatments to study the influence of abiotic stress on the expression of *BcTSA*. The aerial parts were sprayed with a solution containing either 100 μM MeJA, 100 μM ABA, or 100 μM SA, respectively. The leaves were collected at 0, 2, 4, 6, 8, 12, and 24 hours following treatment and rapidly frozen in liquid nitrogen before being kept at -80°C. Water was used to mock-treat control plants. Three to five leaves from different plants were combined together to make a biological repeat, and three biological repetitions were performed.

### Total RNA isolation and first-strand cDNA synthesis

Following the manufacturer’s instructions, 100 mg of *B. cusia* samples were used to extract total RNA using the Column Plant Total RNA Kit (TransGen, Beijing, China). RNA samples were tested for quality and concentration using ethidium bromide-stained agarose gel electrophoresis and spectrophotometer measurement on a NanoDrop 2000C Spectrophotometer (Thermo Scientific, Waltham, MA, USA). For cDNA synthesis, samples having an optical density absorption ratio at OD260/280 between 1.9 and 2.2 and an OD260/230 more than 2.0 were employed. TransScript One-Step gDNA Removal and cDNA Synthesis SuperMix (TransGen, Beijing, China) was used to create first-strand cDNA from 1 μg of total RNA, according to the manufacturer’s instructions.

### Isolation and characterization of BcTSA

The full length of the *BcTSA* ORF sequence was derived from the *B. cusia* transcriptome database. The coding sequences of the *BcTSA* gene was amplified by PCR from cDNA with *pfu* DNA polymerase (New England Biolabs, Ipswich, MA, USA) using the gene-specific primers BcTSA-F and BcTSA-R (**Table S1**). The targeted fragments were purified with the EasyPure Quick Gel Extraction Kit (TransGen, Beijing, China) and cloned into the T-vector with the pEASY™-Blunt Zero Cloning Kit (TransGen, Beijing, China). The generated plasmids were transformed into *E. coli* DH5α cells and selected by means of antibiotic resistance. The sequences were validated by DNA sequencing via Sangon (Shanghai, China).

### Bioinformatics analysis of BcTSA

To identify the whole ORF of *TSA* sequences, NCBI ORF Finder (http://www.ncbi.nlm.nih.gov/orffinder/) and Vector NTI Advance™ 11.0 were used. The deduced amino acid sequences were examined using Vector NTI Advance software to calculate the theoretical isoelectric point (pI) and molecular weight (MW) of BcTSA protein. PSORT (http://psort.hgc.jp/) was used to predict the subcellular localization of the BcTSA protein. To get the amino acid sequences of TSAs different from plant species, a keyword search of the National Center for Biotechnology Information database-NCBI (https://www.ncbi.nlm.nih.gov) was undertaken. The deduced polypeptide sequence of BcTSA was aligned with those of SsTSA (TEY16427), SiTSA (XP_011082598), HiTSA (PIN15896), PbTSA (XP_009354864), MdTSA (XP_008382958), PmTSA (XP_008235332), PyTSA (PQQ06723), FvTSA (XP_004289899), CsTSA (XP_028076212), LgIGL (ACJ02772) and AsIGL (ACJ02768) by Clustal X v2.1. The phylogenetic tree was constructed using the Neighbor-Joining method with MEGA 5.0 software (The Biodesign Institute, Tempe, AZ, USA). The SOPMA tool (https://npsa-pbil.ibcp.fr/cgi-bin/npsaautomat.pl?page=npsasopma.html) was utilized to predict the secondary structure and functional domains of the BcTSA protein. The Simple Modular Architecture Research Tool (SMART, http://smart.embl-heidelberg.de/) was used to identify the conserved sequence domains of BcTSA. The three-dimensional structure of BcTSA was modeled using Swiss-Model (http://swissmodel.expasy.org/) and PyMOL (https://www.pymol.org/2/).

### Real-time quantitative PCR

The real-time quantitative PCR (RT-qPCR) mixture contained 1.0 μL cDNA template, 10 μL 2 × master mix, 1.0 μL forward primer (10 μM), 1.0 μL reverse primer (10 μM), and 7 μL RNase-free ddH_2_O and was performed on a Thermal Cycler Dice Real Time System TP800 (Takara, Tokyo, Japan) according to the instructions of SYBR PreMix Ex Taq (Takara Bio, Dalian, China). The expression levels were normalized with the *18S* control gene using the 2^-ΔΔCt^ method (**Table S1**) (Huang et al., 2017). All RT-qPCR experiments were performed in triplicate.

### Subcellular localization of BcTSA

To create BcTSA-GFP, the ORF of *BcTSA* was fused to the pCAMBIA1301-GFP vector using *Bgl* II and *Spe* I restriction sites. The primers used for subcloning are listed in **Table S1**. DNA sequencing validated the sequence and fusion of GFP under the control of the cauliflower mosaic virus (CaMV) 35S promoter. Using the PEG-mediated transfection technique, the BcTSA-GFP fusion protein was transiently expressed in freshly produced rice protoplasts. Transformed protoplasts were incubated overnight at room temperature (23-25°C), and transient expressions were seen using a confocal laser scanning microscope (Nikon, Tokyo, Japan). The GFP fluorescence was recorded at excitation wavelengths of 488 nm and emission wavelengths of 505-530 nm. A wavelength longer than 650 nm was used to capture the red autofluorescence caused by chlorophylls as previously described (Tan et al., 2015).

### Complementation of *E. coli ΔtrpA* mutant with the *BcTSA* cDNA

The complementation assays were performed essentially as described with some modifications (Zhang et al., 2008). The *E. coli ΔtrpA* mutant strain CGSC 9129, which contains an interruption/deletion of the *trpA* gene, was purchased from Yale University’s *E. coli* Genetic Stock Center. The ORF of *BcTSA* was cloned in pBAD-TOPO (Invitrogen, Carlsbad, CA, USA) expression vector for functional expression in *E. coli* using specific primers specified in **Table S1** according to the manufacturer’s instructions. The untransformed *E. coli* mutant strain was cultured in LB medium with kanamycin at a concentration of 50 mg/mL. All strains were cultivated on M9 basic medium, supplemented with 1M MgSO4, 1M CaCl2, 20% glucose, glycerol, 100 mg/mL of ampicillin, and 20% arabinoses for inducible expression of BcTSA, then cultured at 37°C in the dark for 2 d. The untransformed strains or transformed strains containing the pBAD-TOPO vector serve as a negative control. The medium supplemented with 100 mg/mL tryptophan was used as a positive control.

### Construction of *BcTSA* overexpression vector and *I. indigotica* transformation

The whole coding sequence of *BcTSA* was amplified from leaf cDNA using primers PHB-TSA-F and PHB-TSA-R (**Table S1**). The fragment was subsequently inserted into the *Bam* HI and *Spe* I restriction sites of the modified binary vector PHB-flag, which was driven by two 35S promoters. A*grobacterium tumefaciens* C58C1 transmitted the final construct to *I. indigotica* leaf explants. The hairy root cultures were created by transforming *A. rhizogenes* C58C1 as previously reported (Ma et al., 2017). After 45 days of culturing, the hairy roots were harvested for DNA and RNA extraction, as well as metabolite analyses. In this research, at least three separate lines were evaluated for gene expression and metabolism.

### PCR identification of transgenic hairy roots

The CTAB technique was used to extract genomic DNA from transgenic hairy roots after 45 days of cultivation. To determine the presence of inserted *BcTSA* fragments in *BcTSA* overexpressed lines, the primers PHB-TSA-F and *rbcsr* based on the sequence of vector PHB-flag were utilized. In *A. tumefaciens* strain C58C1, *rolB* and *rolC* represent DNA fragments of T-DNA in *Ri*, and *hpt* represents the hygromycin resistance gene of PHB-flag, indicating that PHB-flag: BcTSA has been effectively transformed into hairy roots. The expression levels of *BcTSA* genes in all positive lines were examined using RT-qPCR. All primers are listed in Supplementary **Table S1**. At least three separate control lines were evaluated in these studies, with the mean value serving as the control.

### Extraction and quantification of indole alkaloids by LC-MS/MS

Hairy roots of *I. tinctoria* were dried at 50°C and pestled into fine powder. 0.2 g of samples were extracted with 5 mL of methanol: chloroform (1:1 vol). The suspension was supersonic for 30 min and then centrifuged at 10 000 rpm for 15 min to remove the suspended particles. The supernatant was transferred and the powder was extracted with 5 mL of reagents again (Yu et al., 2019). The final supernatant was filtered through a 0.22 μm organic membrane. Five millilitres of the extracting solution was evaporated to dryness and redissolved in 200 μL methanol.

LC-MS/MS analysis was run on an Agilent 1200 infinity LC column coupled with an Agilent 6410 Triple Quadrupole LC/MS System (Agilent, USA). The LC operating conditions were as follows: LC column, Poroshell 120 EC-C18, 3.0 μm×150 mm, 2.7 μm; mobile phase, 5 mM ammonium acetate and 0.2% solution of formic acid (A phase) and B phase consisted of a mixture of acetonitrile and methanol (50:50 v/v) with 5 mM ammonium acetate; total flow rate of mobile phase, 0.3 mL/min; total run time including equilibration, 26 min. The injection volume was 5 μL. The MS/MS was operated with an electrospray ionization source in positive ion mode with multiple reaction monitoring. The nebulizer gas pressure was set at 40 psi with a source temperature of 350°C and gas flow at 10 L/min. The capillary voltage was 4 000 V (positive mode). High-purity nitrogen gas was used as collision cell gas. The raw chromatograph and mass spectrogram data were processed with the MassHunter Workstation software (Agilent, USA).

### Statistical analysis

Experiments were performed in triplicate, and are presented as mean ± standard error of the mean (SEM) calculated by GraphPad Prism 8.0 software. A Student’s *t*- test was used to compare two groups. One-way ANOVA followed by the Dunnett *post hoc* test was used for multiple comparisons versus the control group. *p* < 0.05 and *p* < 0.01 were set as the criterion for statistical significance.

## Results

### Cloning and bioinformatics analysis of BcTSA

**Figure 2:**
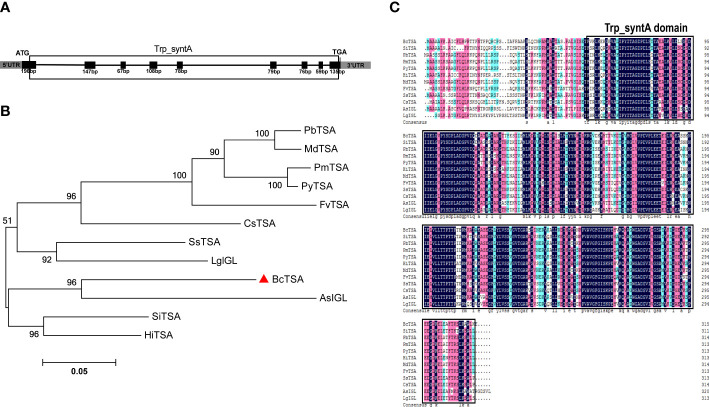
Molecular identification and phylogenetic analysis of *BcTSA*. **(A)** A schematic representation of the exon and intron organization of *BcTSA*. Black boxes indicate exons, intervening lines indicate introns. **(B)** Phylogenetic tree of BcTSA and other plant TSA proteins using MEGA 5.0 software based on the neighbor-joining method. **(C)** The homology comparison of amino acid alignment of TSA in 12 plant species. The Trp_syntA domain is boxed.

RT-PCR was used to isolate the full-length genomic and cDNA sequences of *BcTSA* from *B. cusia*. The full-length genomic *BcTSA* gene was 4381 bp in size and consisted of 8 introns and 9 exons (**Figure 2A**). The cDNA of *BcTSA* is composed of 948 bp and encodes a putative alpha-tryptophan protein of 315 amino acids. The predicted protein had a calculated MW of 33.545 kDa with a pI of 5.61. According to amino acid analysis, the BcTSA putative protein featured a conserved Trp_syntA domain at the 57th-314th amino acids, which is similar to TSAs found in other plants. The secondary structure estimation revealed that the BcTSA peptide consisted of 43.49% alpha helices, 17.14% extended strands, 6.67% beta twists, and 32.7% random coils. The most frequent structural features were alpha helices and random coils, which penetrated much of the BcTSA secondary structure, whereas extended strands and beta turns were scattered throughout the protein. The 3D structure of BcTSA was simulated by the SWISS-MODEL server using the crystal structure of Tryptophan synthase alpha chain homolog BX1 (PDB id: 1rd5.1) as a template with the consistency of predicted results at 59.46%, implying that it contains the Tryptophan synthase alpha-subunit functional domain.

To discover more regarding the phylogenetic relationship between BcTSA and its close homologs, a rooted phylogenetic tree was built using the amino acid sequences of BcTSA from *B.cusia* and 11 other closet homologs (including IGL from *Lamium galeobdolon* and *Aphelandra squarrosa*, as well as contain Trp_syntA domain) from 11 angiosperms (**Figure 2B**). The phylogenetic tree showed BcTSA was structurally closely related to AsIGL in *Aphelandra squarrosa* (**Figure 2B**), the two plant species of which belonged to the Acanthaceae family, suggesting a close phylogenetic relationship. BcTSA also shared 68-78.15% amino acid similarity with other IGL or TSA proteins from other species, according to multiple sequence alignment (**Figure 2C**).

### Expression profile and subcellular localization

**Figure 3:**
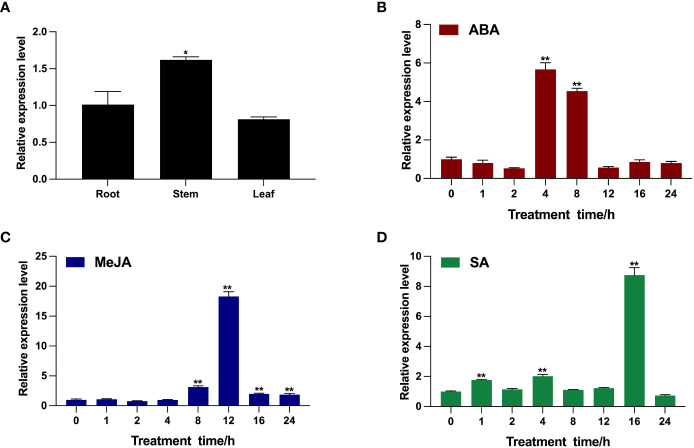
Transcription pattern of *BcTSA* in different *B*. *cusia* tissues **(A)** and under the induction of phytohormones (ABA **(B)**, MeJA **(C)**, and SA **(D)**) by RT-qPCR analysis. Data are represented as the mean ± SEM; *P < 0.05 and, **P < 0.01 compared to the control group. All reactions were carried out in triplicate, and each experiment was repeated twice.

To characterize the transcription pattern of *BcTSA* in *B. cusia*, the transcription level of *BcTSA* was analyzed by RT-qPCR. As shown in **Figure 3A**, the *BcTSA* transcript was detected in all analyzed tissues of *B. cusia*, with the highest transcript levels found in the stems, followed by the roots, and the lowest levels found in the leaves. The RT-qPCR showed the transcription level of *BcTSA* rose dramatically under the stress of ABA, SA, and MeJA with significant variations depending on the time and/or the phytohormones (**Figures 3B–D**). During the ABA treatment, the transcript level of *BcTSA* decreased gradually for the first two hours, then rapidly rose to a peak at 4 h (5.66-fold), and then downregulated (**Figure 3B**). After treatment with MeJA, *BcTSA* expression decreased modestly for 2 hours before rapidly increasing. The highest level of *BcTSA* expression was observed after 12 h of 0.1 mM MeJA treatment, with an expression of approximately 18.29 times higher than control levels (**Figure 3C**). The transcript of *BcTSA* rapidly increased 1.77-fold after SA treatment for 1 h and then declined after 2 h of treatment, but again increased up to 2-fold at 4 h of treatment. Along with the extension of treatment time, the transcript level of *BcTSA* reached its highest point at 16 h, with an increase of ~8.75-fold, and subsequently dropped (**Figure 3D**).

**Figure 4:**
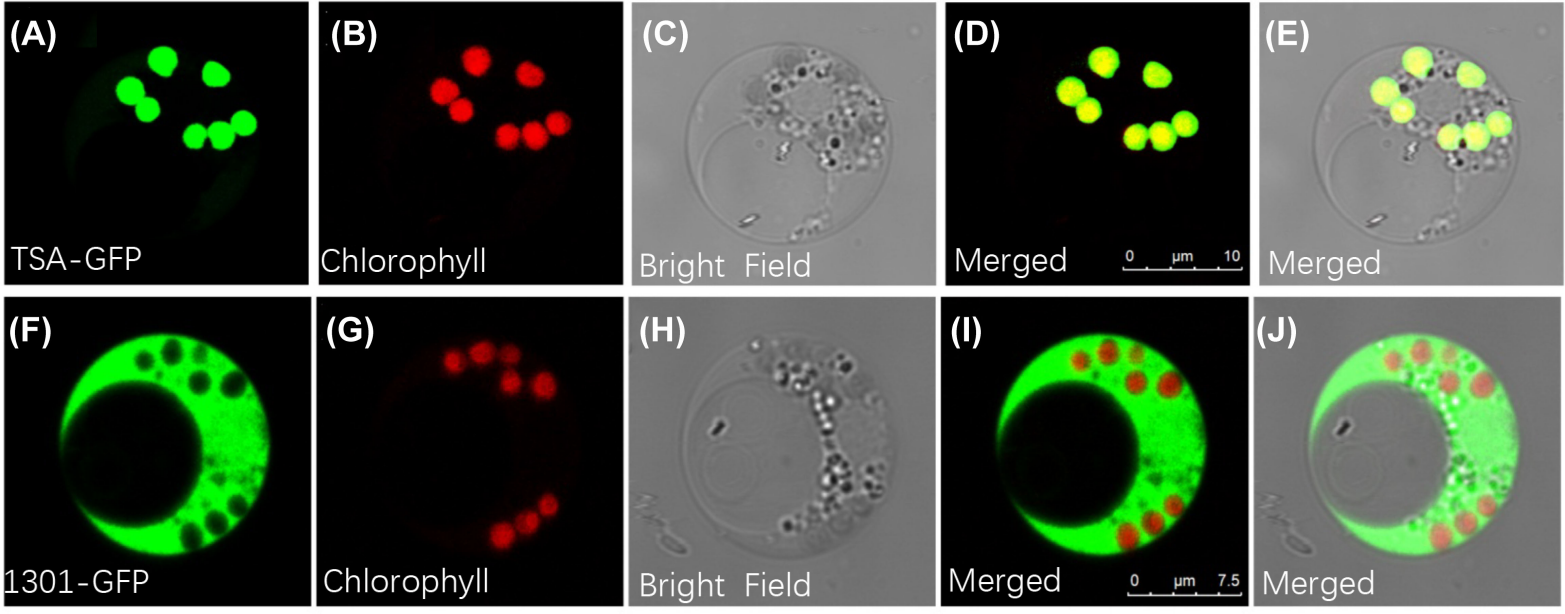
Subcellular localization of BcTSA-GFP transiently expressed in rice protoplast cells. **(A, F)** GFP fluorescence; **(B, G)** Red chlorophyll autofluorescence in chloroplasts; **(C, H)** Optical fields; **(D, I)** The merged signal of **(A, B)** or **(F, G)**; **(E, J)** The merged signal under bright fields.

Given the possibility that the indole alkaloids route exists as a metabolic channel connected with chloroplasts, it was important to identify BcTSA’s subcellular location. As shown in **Figure 4A-E**, when the GFP fusion proteins of BcTSA were constitutively expressed in rice protoplast, their fluorescent signals merged nicely with the chlorophyll autofluorescence from chloroplasts, demonstrating that the BcTSA protein could be localized to the chloroplasts. In contrast, free GFP was discovered uniformly throughout the cytoplasm (**Figure 4F–J**).

### BcTSA encodes a functional protein catalyzing the formation of indole

**Figure 5:**
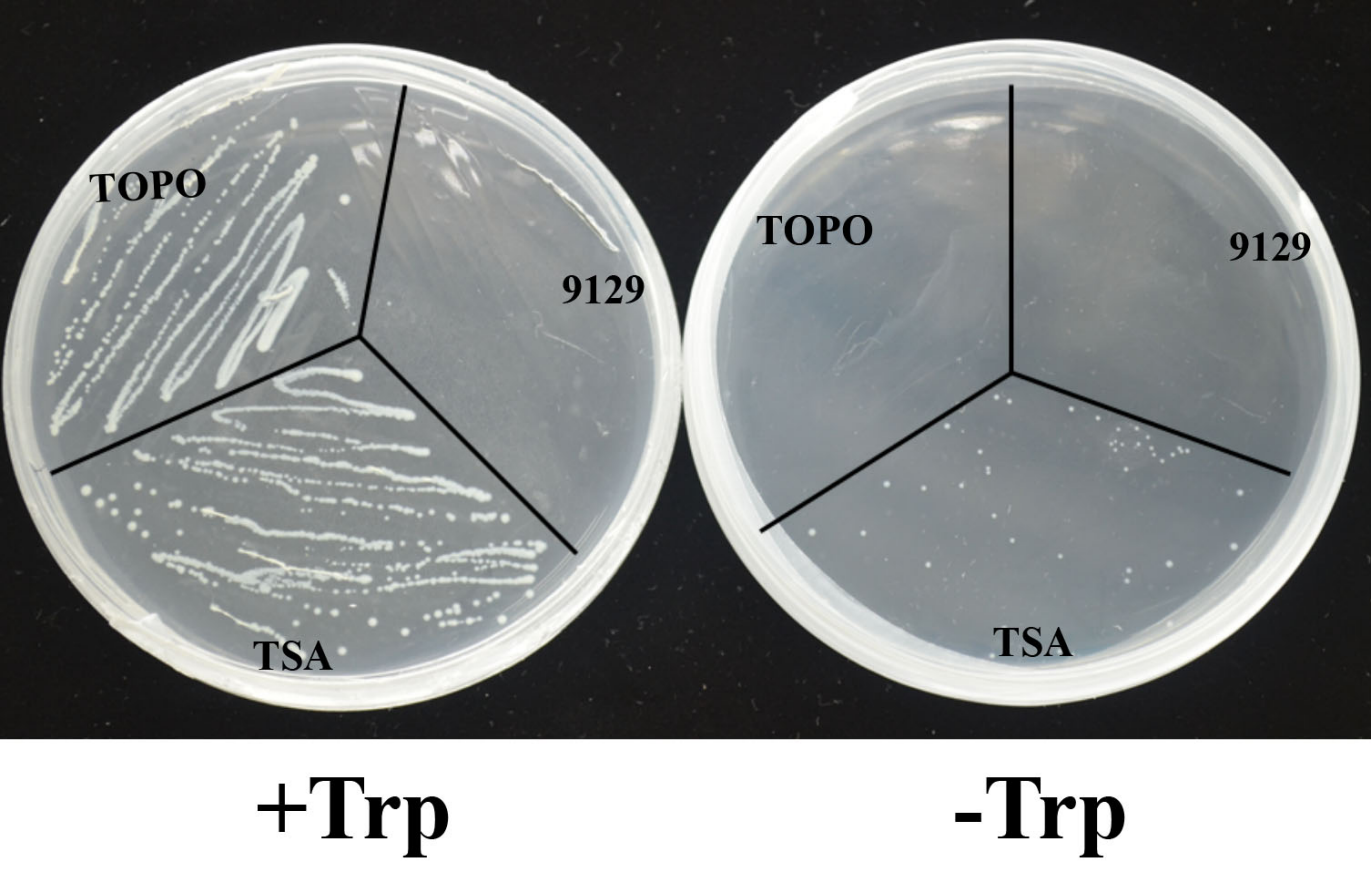
Complementation of *E.coli △trpA* mutant with the *BcTSA* cDNA. (9129) *E.coli △trpA* mutant. (TOPO) transformant containing the pBAD-TOPO vector. (TSA) transformant containing the *BcTSA* ORF in the pBAD-TOPO vector.

To determine whether BcTSA has the enzyme activity that synthesizes the production of indole from IGP, the recombinant expression plasmid pBAD-TOPO for BcTSA was used to transform into the *E. coli* (9129) mutant strain *ΔtrpA*, which is a Trp-auxotrophic mutant with a deleted *TSA* gene. In contrast to the control transformant TOPO, which only contains the pBAD-TOPO vector, *E. coli* (TSA) colonies transformed with the *BcTSA* cDNA were able to survive on M9 basic minimal medium containing 0.02% arabinose without tryptophan. The presence of *BcTSA* cDNA fragments in plasmids isolated from transformant colonies growing without tryptophan were determined by PCR using *BcTSA* specific primers (**Table S1**). The observation suggests that *BcTSA* can restore the *E. coli ΔtrpA* mutant (**Figure 5**). As a consequence, we conclude that BcTSA catalyzes indole synthesis in *B. cusia*.

### Overexpression of *BcTSA* affected the production of indole alkaloids in *I. indigotica*

**Figure 6:**
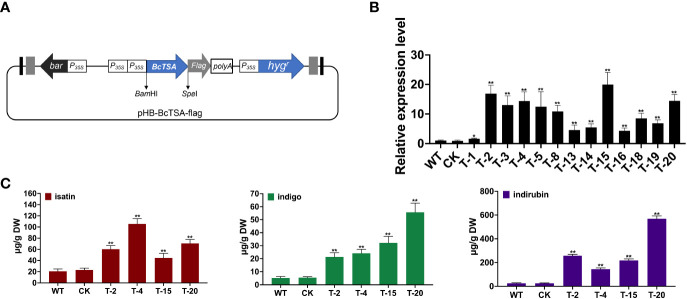
Hairy root cultures of BcTSA-overexpressed in *l. indigotica*. **(A)** Schematic representation of the overexpression vector used for hairy root establishment. **(B)** *BcTSA* transcript abundance in hairy root cultures by RT-qPCR. **(C)** LC-MS/MS analysis of isatin, indole, and indirubin of different *I. indigotica* hairy root including the *BcTSA* overexpression lines, transformed with the empty vector, and the WT control. Each data point is the average of three biological replicates. Data are represented as the mean ± SEM; *P < 0.05 and, **P < 0.01 compared to the WT group.

The PHB-BcTSA construct was created by successfully subcloning the full-length *BcTSA* cDNA without a stop codon into the plasmid PHB-flag utilizing the *Bam*HI and *Spe*I restriction sites (**Figure 6A**). The transcript levels of endogenous *BcTSA* were examined by RT-qPCR in positive lines (named T). The expression level of *BcTSA* was massively increased in the positive transgenic lines, with 16.89-, 19.95-, and 14.43-fold enhancements in T-2, T-15, and T-20 compared to WT plants, according to RT-qPCR data (**Figure 6B**). Four separate lines (2, 4, 15, 20) with higher *BcTSA* transcript levels were chosen from all of the independent hygromycin-resistant transgenic *I. indigotica* hairy roots harboring the PHB-BcTSA construct. LC-MS/MS analysis revealed that the concentration of indole alkaloids in four different BcTSA-overexpressed *I. indigotica* hairy root lines was considerably enhanced compared to the WT group (**Figure 6C** and **Figure S1**). The T-4 line with the maximum isatin concentration (105.58 μg/g DW), followed by the T-20 and T-2 lines, which were 5.1-, 3.4-, and 2.9-fold higher than that in its WT (20.79 μg/g DW) counterparts, respectively. Line T-20 showed the greatest level of indigo and indirubin, at 55.73 μg/g DW and 568.47 μg/g DW, respectively, which increased 10.82-fold and 22.45-fold when compared to the WT. This observation demonstrated BcTSA’s beneficial properties in the metabolic engineering of indole alkaloids synthesis.

## Discussion

Alkaloids are greater beneficial substances in Chinese herbal medicines because they are a family of natural nitrogen-containing secondary metabolites with substantial biological activity (Parr et al., 2015). Indole alkaloids are a variety of alkaloids distinguished by the presence of an indole structural element (Huang et al., 2016). There is growing evidence that the levels of indole alkaloids in *B. cusia* are higher than in other plants such as *I. indigotica*, *P. tinctorium*, and *Indigofera tinctoria*, and that there is a great disparity in the contents of indoxyl beta-D-glycoside, indigo, and indirubin between organs (Liau et al., 2007). Additionally, *B. cusia* is capable of serious environmental tolerance as well as fairly rapid growth through clonal multiplication, demonstrating its promise as a resurgent natural indigo and indirubin source (Xu et al., 2020).

The indigo and indirubin synthesis, which follows the indole pathways (**Figure 1**), produces indole as the first product (Zhang et al., 2022). The biosynthetic precursor of indigo in higher plants was identified 25 years ago as indole that did not originate from tryptophan (Jin et al., 2016). Indole biosynthesis has a direct impact on indigo and indirubin levels. In this study, the *BcTSA* gene was extracted and identified from the *B. cusia* transcriptome data, and the structure, properties, and subcellular localization of the putative protein were predicted by bioinformatic tools. Bioinformatics can produce more trustworthy results in less time and at a lower cost than traditional laboratory-based experimental research. The findings of phylogenetic tree analysis indicated that BcTSA exhibited high similarity to orthologues in other plants, indicating that the TSA enzyme was highly conserved over its evolutionary process (**Figure 2A**).

RT-qPCR results showed that the *BcTSA* gene is constitutively expressed in all examined tissues, with the highest levels in stem tissues and the lowest levels in leaves (**Figure 3A**), a pattern similar to that of the *TSA* gene in *A. thaliana* (Zhang et al., 2008) and *Isatis tinctoria* (Salvini et al., 2008). Given that the leaves and stems are the main organs for indole alkaloids accumulation in *B. cusia*, it is worth noting that the transcription level of *BcTSA* displayed similar expression patterns in different tissues, with no significant difference in roots and leaves (p > 0.05); thus, we speculate that the above results will not affect the function of *BcTSA* in indole alkaloids biosynthesis. Abundant research have been conducted to explore the expression patterns of indole alkaloids biosynthesis genes under elicitor treatments, which will aid in the discovery of molecular induction mechanisms for future indole alkaloids biosynthesis engineering in plants (Zhu et al., 2015; Yu et al., 2019). In this investigation, defense-related signaling molecules such as MeJA, SA, and ABA were used to analyze the expression profile of *BcTSA*. The results showed that the expression of *BcTSA* had a prominent diversity to that of control and may play a vital role in response to abiotic stresses (**Figure 3**). As well-known, exogenous MeJA treatment significantly boosted the concentration of indole alkaloids (Abouzeid et al., 2017; Lin et al., 2019), it is tempting to speculate that the expression level of *BcTSA* was likely to be little correlation with indole alkaloids synthesis.

Although the regulatory mechanisms that indole alkaloids biosynthesis are poorly understood, there is ample evidence that TSA catalyzes the first committed step of the chloroplast metabolic pathway of indole synthesis, namely the converts IGP to indole, which occurs predominantly in the chloroplasts (Radwanski and Last, 1995; Kriechbaumer et al., 2008; Zhang et al., 2022). Furthermore, the precursors of indigo and indirubin, indoxyl and indole, are frequently generated in the cytosol or chloroplasts. We also confirmed that BcTSA is localized to chloroplasts by examining the expression of a GFP fusion protein in transfected rice cells (**Figure 4**), which was consistent with previous findings in *A. thaliana* and maize (Kriechbaumer et al., 2008; Zhang et al., 2008), indicating a role in indole alkaloids biosynthesis. More crucially, we found BcTSA may complement the auxotrophic phenotype of an *E. coli ΔtrpA* mutant without tryptophan (**Figure 5**), indicating that BcTSA is a functional protein that can catalyze indole synthesis. Taken together, our findings suggest that BcTSA might be positively correlated with the synthesis of indigo and indirubin.

Indole alkaloids, such as indican, indigo, and indirubin, are important active components in *B. cusia* (Gu et al., 2014; Hu et al., 2015; Feng et al., 2016; Xu et al., 2021). As *B. cusia* is a perennial plant and stable transformation is difficult and time-consuming, we utilized *I. indigotica* as an alternate method of assessing *BcTSA* function since it is an essential model plant for researching the biosynthesis route of indole alkaloids. Overexpression of *BcTSA* enhanced the accumulation of isatin, indigo, and indirubin in *I. indigotica* hairy roots compared to WT lines (**Figure 6**). Considering molecular oxygen was a massive environmental component in the regulation of indole alkaloids synthesis, we did not observe a perfectly positive association between the increment in indole alkaloids content and the change in *BcTSA* transcript level. To sum up, these findings show that *BcTSA* plays a crucial role in the accumulation of indole alkaloids in *B. cusia*, which is consistent with the expected regulatory activities. Gene manipulation of the indole pathway can effectively change the metabolic level of the target pathway, and may also cause changes in the metabolic flow of the whole indole alkaloids pathway network. Consequently, the molecular catalytic properties of *BcTSA* in indole alkaloid biosynthesis in *B. cusia* revealed here will give a novel perspective for functional research of the indole alkaloids pathway.

## Conclusions

In this work, the full-length gDNA and cDNA sequences of the *BcTSA* gene from *B. cusia* were successfully cloned and molecularly characterized. *BcTSA* was up-regulated under phytohormone treatments, suggesting that *BcTSA* is involved in the signal transduction pathway of biotic stresses. The recombinant BcTSA displayed the ability to catalyze the formation of indole from IGP. Overexpression of *BcTSA* definitively promoted an increased accumulation of indole alkaloids in hairy root cultures of *I. indigotica*. Our findings contribute new molecular insights for future studies regarding indole alkaloids biosynthesis and hold considerable promise for improving *B. cusia* quality through the genetic engineering of critical genes in molecular breeding.

## Data availability statement

The original contributions presented in the study are included in the article/**Supplementary Material**. Further inquiries can be directed to the corresponding authors.

## Author contributions

ZG, JC, ZL, HT, LZ and YD conceived the study. ZG and YH performed the experiments; ZG, JC, ZL, YH, HT, LZ and YD analyzed the data. ZG, JC, HT, LZ and YD wrote the paper. HT, LZ and YD revised the paper. All authors contributed to the article and approved the submitted version.
