# Supplementary material for: Molecular cloning and functional characterization of BcTSA in the biosynthesis of indole alkaloids in Baphicacanthus cusia
Source: Front Plant Sci. 2023 Apr 17;14:1174582. doi: 10.3389/fpls.2023.1174582 (PMC10149986; doi:10.3389/fpls.2023.1174582)
Supplement: Supplementary file 1 [file DataSheet_1.docx]

**TABLE S1 |** List of all primers used in this study.

| ***Primers*** | ***Function*** | ***Primer Sequences (5’-3’)*** |
| --- | --- | --- |
| BcTSA-F | Gene clone | CCTTTCATTCTTTCAGAGCAGC |
| BcTSA-R | Gene clone | GAAGACAACTGATGGCAGCT |
| qPCR18S-F | RT-qPCR | GCTTCCCTCCCGACAATTTC |
| qPCR18S-R | RT-qPCR | AGTCGGGTTGTTTGGGAATG |
| qPCRTSA-F | RT-qPCR | ACCGGAGTTCATGGACTTGT |
| qPCRTSA-R | RT-qPCR | AGCCGGGACCCTTTCATTTA |
| subTSA-F | Subcellular localization | AAAGATCTTATGGCAGCTGCCGCTTTCAA (*Bgl*II) |
| subTSA-R | Subcellular localization | AAACTAGTTTCTTTCAGAGCAGCTTTTA (*Spe*I) |
| pBAD-TSA-F | Complementation  Assay | GAGGAATAATAAATGGCAGCTGCCGC |
| pBAD-TSA-R | Complementation  Assay | TCATTCTTTCAGAGCAGCTTTTAAGC |
| PHB-TSA-F | Overexpression construction | AAAGGATCCATGGCAGCTGCCGCTTTCAA (*Bam*HI) |
| PHB-TSA-R | Overexpression  construction | AAAACTAGTTTCTTTCAGAGCAGCTTTTA (*Spe*I) |
| rbcsr | PCR | ATTAACTTCGGTCATTAGAGGC |


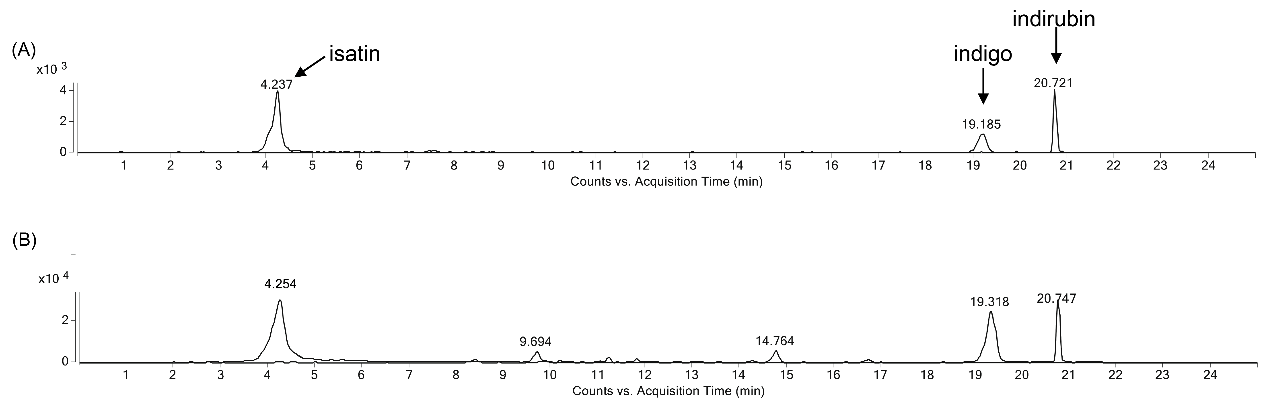


**FIGURE S1 |** LC-MS/MS analysis of *TSA* transgenic hairy roots of *I. indigotica*. The extracted peak identification: **(A)** Standards, **(B)** The result of T-2 transgenic line.
